# Nomograms for predicting difficult airway based on ultrasound assessment

**DOI:** 10.1186/s12871-022-01567-y

**Published:** 2022-01-13

**Authors:** Bin Wang, Weidong Yao, Qi Xue, Mingfang Wang, Jianling Xu, Yongquan Chen, Ye Zhang

**Affiliations:** 1 Department of Anesthesiology, The Second Affiliated Hospital of Anhui Medical University, 678# Furong Road, Hefei, Anhui Province China; 2 Department of Anesthesiology, The First Affiliated Hospital of Wannan Medical College, Yijishan Hospital, Wuhu, China

**Keywords:** Airway management, Intubation, Intratracheal, Laryngoscopy, Nomogram, Ultrasonography

## Abstract

**Background:**

Accurate prediction of the difficult airway (DA) could help to prevent catastrophic consequences in emergency resuscitation, intensive care, and general anesthesia. Until now, there is no nomogram prediction model for DA based on ultrasound assessment. In this study, we aimed to develop a predictive model for difficult tracheal intubation (DTI) and difficult laryngoscopy (DL) using nomogram based on ultrasound measurement. We hypothesized that nomogram could utilize multivariate data to predict DTI and DL.

**Methods:**

A prospective observational DA study was designed. This study included 2254 patients underwent tracheal intubation. Common and airway ultrasound indicators were used for the prediction, including thyromental distance (TMD), modified Mallampati test (MMT) score, upper lip bite test (ULBT) score temporomandibular joint (TMJ) mobility and tongue thickness (TT). Univariate and the Akaike information criterion (AIC) stepwise logistic regression were used to identify independent predictors of DTI and DL. Nomograms were constructed to predict DL and DTL based on the AIC stepwise analysis results. Receiver operating characteristic (ROC) curves were used to evaluate the accuracy of the nomograms.

**Results:**

Among the 2254 patients enrolled in this study, 142 (6.30%) patients had DL and 51 (2.26%) patients had DTI. After AIC stepwise analysis, ULBT, MMT, sex, TMJ, age, BMI, TMD, IID, and TT were integrated for DL nomogram; ULBT, TMJ, age, IID, TT were integrated for DTI nomogram. The areas under the ROC curves were 0.933 [95% confidence interval (CI), 0.912–0.954] and 0.974 (95% CI, 0.954–0.995) for DL and DTI, respectively.

**Conclusion:**

Nomograms based on airway ultrasonography could be a reliable tool in predicting DA.

**Trial registration:**

Chinese Clinical Trial Registry (No. ChiCTR-RCS-14004539), registered on 13th April 2014.

**Supplementary Information:**

The online version contains supplementary material available at 10.1186/s12871-022-01567-y.

## Background

In the operating room, intensive care unit, and emergency department, the incidence of difficult airway (DA) is high, threatening the lives of patients [1–6]. DA is defined as difficulty in establishing artificial ventilation under general anesthesia and emergency conditions, which has been widely studied [7–11]. Even though the assessment of DA is necessary before intubation, the predictive ability of existing methods is very limited and cannot meet clinical needs, because of many affecting factors [4–6]. Thus, improving the DA prediction methods has great clinical significance.

At present, methods such as interincisor distance (IID), thyromental distance (TMD), modified Mallampati test (MMT) score, and upper lip bite test (ULBT) score are mostly used in clinical practice [12]. However, these indicators have limited performance, low sensitivity and specificity, and low positive predictive values [13, 14]. Recent studies have suggested that anatomical measurement of upper airway using ultrasonography could improve the DA prediction performance [15–18]. For examples, by use of ultrasonography, temporomandibular joint (TMJ) mobility [19] and tongue thickness (TT) [20] have been successfully used to predict DAs. However, DA associates with the whole anatomy of upper airway structure, and a single indicator is not sufficient to reflect the functionality of upper airway and cannot accurately predict DA. To overcome the predicting inability of single factor, some studies combined multiple indicators to improve DA prediction, for examples, the “3–3-2” rule [21], modified “look, evaluate, Mallampati score, obstruction, and neck mobility” (LEMON) criteria [22], and “Wilson” scores [23]. Although the combination of multiple indicators improves the sensitivity of DA prediction to a certain extent, at the same time these methods also reduce the specificity of DA prediction. Furthermore, the simple data processing method, ignoring the numerical size, has certain limitation in dealing with complicated clinical conditions, which may lead to loss of valuable information. Even in ultrasonography-guided methods, the absence of valuable information is still a major drawback of DA risk prediction.

Of the available risk assessment models, the nomogram predictive model combines the parameters by endowing different weights, and provides a highly accurate, evidence-based, individualized risk estimation, thus facilitating clinical decision-making [24–28]. Therefore, we proposed a hypothesis that nomogram can improve the prediction ability of difficult tracheal intubation (DTI) and difficult laryngoscopy (DL). In this study, using a clinical trial data, we established a nomogram model for DA prediction, which could significantly improve the DA prediction and reduce the harm of DA.

## Methods

All participants were from a prospective observational DA study conducted at the Yijishan Hospital of Wannan Medical College, which has been described previously [20, 29]. In brief, the study enrolled 2254 patients who underwent selective surgery and general anesthesia with endotracheal intubation from May 2014 to May 2016 and aimed to explore risk factors of DTI and DL. The study was approved by the ethics committee of the First Affiliated Hospital of Wannan Medical College [No. (2013) 91] and registered at the China Clinical Trial Registration Center (Registration No. ChiCTR-RCS-14004539). Written informed consent was obtained from the patients before enrollment. All methods were performed in accordance with the relevant guidelines and regulations.

1. Inclusion and exclusion criteria:

Patients undergoing the elective operation, general anesthesia and tracheal intubation who were aged > 18 years old and had an American Society of Anesthesiologists (ASA) grade of I-III were included. We excluded patients with a confirmed history of DA or maxillofacial trauma and cervical spine injury; patients with maxillofacial, orolingual, or cervical tumors; patients with subglottic stenosis affecting tracheal intubation; patients with nasotracheal intubation or double-lumen endotracheal intubation; and patients with incomplete clinical information.

2) Airway assessment method

Classical predictors of DA:

Before the induction of general anesthesia, the IID, MMT score, ULBT score, TMD, height and weight of patients were evaluated or measured. Evaluation using classical methods was conducted by specialist staff, who with more than five years of clinical experience in performing intubations and airway assessment.

Measurement of TMJ mobility by ultrasonography [19]:

TMJ mobility was measured before anesthesia by a high-frequency linear array probe placed in front of the ear. The patients were asked to open and close their mouths, and the distance of anterior and inferior sliding of the mandibular condyle was measured dynamically.

Measurement of TT by ultrasonography [20]:

The patients were guided in the supine position, the head was stretched, the sniffing position was held, and the tongue was relaxed. A low-frequency convex array probe was placed in the middle sagittal plane of the chin; one end was placed flat in the middle of the mandible, and the other end was pointed at the thyroid cartilage. The direction of the probe was adjusted to be perpendicular to the skin. The distance between the submental skin and tongue surface, that is, the thickness of the tongue body, was measured by ultrasound.

The ultrasonography evaluation was conducted by a physician with ultrasound experience at least 40 times training, which was proved with good reliability among different sonographers [19].

3) General anesthesia induction program

The general anesthesia induction program included midazolam 0.02–0.05 mg/kg, fentanyl 3–4 μg/kg, vecuronium 0.1 mg/kg, propofol 1–2 mg/kg. Tracheal intubation was performed after administration of muscle relaxants by experienced anesthesiologists. The patient is in the supine sniffing position. A laryngoscope handle and a Macintosh laryngoscope blade (Sirius XL, Basildon, UK) were used for laryngoscopy. Endotracheal tube type and size were determined according to patient and surgical conditions. After tracheal intubation completed, mechanical ventilation was performed. If a DA is encountered, the anesthesiologist immediately seeks help from the DA management team. The following strategies were provided for difficult airways: video laryngoscope (UE, China)-assisted intubation, laryngeal mask endotracheal intubation or ventilation, fibrebronchoscopy-guided intubation, lighted stylets or light wands, and percutaneous thyrocricocentesis to establish a surgical airway rapidly [20, 29].

4) Study endpoints

The primary endpoint was DTI prediction capability and the secondary was DL prediction capability.

DL was defined as Cormack-Lehane grade III-IV laryngoscopy; that is, when laryngoscopy was performed, only a grade III or grade IV epiglottis could be observed. Cormack-Lehane grade is as follows: Grade I, most of the glottis is visible; Grade II, only the posterior extremity of glottis is visible; Grade III, no glottis can be seen but only the epiglottis; Grade IV, no epiglottis can be seen [30].

DTI was defined as intubation requiring up to three times or an intubation time of more than 10 min.

Endotracheal intubation was performed by anesthesiologists with more than five years of clinical experience who were unaware of the results of the ultrasonographic detection of the DA prediction indicators.

### Statistical analysis

The *χ*^2^ test was used for categorical variables with Bonferroni correction for inter-group comparison. The continuous variables included in this study were skewed distribution by normality test, so Mann-Whitney U-test was used for the continuous variables. Restricted cubic spline (RCS) or the receiver operating characteristic (ROC) curve were used to selected different segmentation criteria for continuous data according to their quantile distribution in positive and negative groups of DL and DTI. And then all the influencing factors after pretreatment were used for the prediction models of DL and DTI respectively. We choose the variables with *P* value less than 0.1 of univariate logistic regression by the Akaike information criterion (AIC) stepwise analysis for the further multivariate logistic regression. Nomograms were constructed to predict DL and DTL based on the multivariate logistic regression results, and the corresponding scorecards were output. Finally, ROC curves were used to evaluate the accuracy of the nomograms. Statistical analyses were conducted with R (version 3.6.0, R Development Core Team) and SAS (version 9.4, SAS Institute Inc., Cary, NC).

## Results

A total of 2254 patients, including 1059 males and 1195 females, were included in this study; 142 (6.30%) patients had DL, and 51 (2.26%) patients had DTI (Table S1). The distribution of continuous variables in DL and DTI was shown in Table S2 and Table S3. Results for RCS and ROC curves for continuous variables segmentation of DL and DTI were presented in Fig. S1, S2, S3, S4, S5, S6, S7, S8 and S9. Univariate logistic regressions of DL and DTI according to RCS were shown in Table S4 and Table S5.

According to the results of univariate logistic regression analysis, ULBT, MMT, sex, TMJ, age, BMI, TMD, IID, and TT were included in multivariate logistic regression analysis. As shown in Table 1, nine variables were considered as independent risk factors for DL, such as ULBT grade III [odds ratio (OR) = 4.114, 95% CI: 2.114–8.005, *P* < 0.001], MMT grade IV (OR = 2.461; 95% CI: 1.411–4.292; *P* = 0.002), TT > 67 mm (OR = 4.525; 95% CI: 1.918–10.672; *P* < 0.001)*.* Five variables were considered as independent risk factors for DTI, such as ULBT grade III (OR = 5.078; 95% CI: 1.451–17.771; *P* < 0.001), TT > 62 mm (OR = 29.451; 95% CI: 6.212–139.622; *P* < 0.001) (Table 2).

**Table 1:** Univariate logistic regression and multivariate logistic analysis showing the association of variables with difficult laryngoscopy

Variable	Univariate logistic regression		Multivariate logistic regression	
	OR (95% CI)	*P*-value	OR (95% CI)	*P*-value
ULBT (grade)				
I	ref.		ref.	
II	2.346 (1.455, 3.784)	<0.001	1.428 (0.812, 2.509)	0.216
III	14.406 (8.447, 24.569)	<0.001	4.114 (2.114, 8.005)	<0.001
MMT (grade)				
I/II	ref.		ref.	
III	1.943 (1.255, 3.007)	0.003	1.016 (0.594, 1.737)	0.955
IV	4.644 (3.077, 7.009)	<0.001	2.461 (1.411, 4.292)	0.002
Sex				
male	ref.		ref.	
female	0.325 (0.223, 0.473)	<0.001	0.285 (0.174, 0.466)	<0.001
TMJ (mm)				
<12	ref.		ref.	
≥12	0.030 (0.018, 0.051)	<0.001	0.044 (0.025, 0.077)	<0.001
Age (y)				
<36	ref.		ref.	
36-51	4.925 (1.512, 16.044)	0.008	2.891 (0.795, 10.521)	0.107
≥52	12.333 (3.885, 39.157)	<0.001	5.113 (1.430, 18.286)	0.012
BMI (kg/m^2^)				
<18.5	ref.		ref.	
[18.5,24)	0.947 (0.504, 1.780)	0.866	1.464 (0.659, 3.250)	0.349
[24,27)	1.325 (0.677, 2.595)	0.411	1.766 (0.726, 4.295)	0.21
[27-30)	1.979 (0.946, 4.138)	0.07	4.172 (1.541, 11.299)	0.005
≥30	0.754 (0.206, 2.753)	0.669	1.587 (0.301, 8.360)	0.586
TMD (mm)				
<65	ref.		ref.	
[65,78)	0.274 (0.182, 0.414)	<0.001	0.391 (0.226, 0.678)	0.001
≥78	0.151 (0.090, 0.254)	<0.001	0.254 (0.130, 0.497)	<0.001
IID (mm)				
<40	ref.		ref.	
≥40	0.181 (0.123, 0.266)	<0.001	0.554 (0.342, 0.897)	0.016
TT (mm)				
<60	ref.		ref.	
60-67	2.561 (1.757, 3.733)	<0.001	1.495 (0.917, 2.436)	0.107
>67	6.985 (3.937, 12.391)	<0.001	4.525 (1.918, 10.672)	0.001

*Abbreviations*: *ULBT* upper lip bite test, *MMT* modified Mallampati test, *TMJ* temporomandibular joint mobility by mandibular condylar mobility measured by ultrasonography, *BMI* body mass index, *TMD* thyromental distance, *IID* interincisor distance, *TT* tongue thickness measured by ultrasonography, *CI* confidence interval

**Table 2:** Univariate logistic regression and multivariate logistic analysis showing the association of variables with difficult tracheal intubation

Variable	Univariate logistic regression		Multivariate logistic regression	
	OR (95% CI)	*P*-value	OR (95% CI)	*P*-value
ULBT (grade)				
I	ref.			
II	4.787 (1.665, 13.766)	0.004	2.614 (0.799, 8.553)	0.112
III	31.143 (10.540, 92.021)	<0.001	5.078 (1.451, 17.771)	0.011
MMT (grade)				
I/II	ref.			
III	1.744 (0.802, 3.794)	0.161		
IV	5.990 (3.082, 11.642)	<0.001		
Sex				
male	ref.			
female	0.435 (0.242, 0.783)	0.006		
TMJ (mm)				
<11	ref.			
≥11	0.009 (0.003, 0.023)	<0.001	0.022 (0.008, 0.061)	<0.001
Age (y)				
<32	ref.			
32-58	3.300 (0.436, 25.000)	0.248	1.156 (0.124, 10.799)	0.899
≥58	11.471 (1.562, 84.230)	0.016	3.150 (0.350, 28.361)	0.306
BMI (kg/m^2^)				
<18.5	ref.			
[18.5,24)	0.789 (0.298, 2.091)	0.634		
[24,27)	0.841 (0.284, 2.490)	0.754		
[27-30)	1.972 (0.649, 5.990)	0.231		
≥30	1.873 (0.436, 8.047)	0.399		
TMD (mm)				
<65	ref.			
≥65	0.302 (0.158, 0.575)	<0.001		
IID (mm)				
<35	ref..			
[35,40)	0.170(0.091,0.320)	<0.001	0.246 (0.110, 0.549)	0.001
≥40	0.023(0.008,0.067)	<0.001	0.109 (0.032, 0.377)	<0.001
TT (mm)				
≤55	ref.			
(55,62]	4.402(1.009,19.208)	0.049	6.251 (1.305, 29.938)	0.022
>62	21.249(5.074,88.994)	<0.001	29.451 (6.212, 139.622)	<0.001

*Abbreviations*: *ULBT* upper lip bite test, *MMT* modified Mallampati test, *TMJ* temporomandibular joint mobility by mandibular condylar mobility measured by ultrasonography, *BMI* body mass index, *TMD* thyromental distance, *IID* interincisor distance, *TT* tongue thickness measured by ultrasonography, *CI* confidence interval

**Fig. 1:**
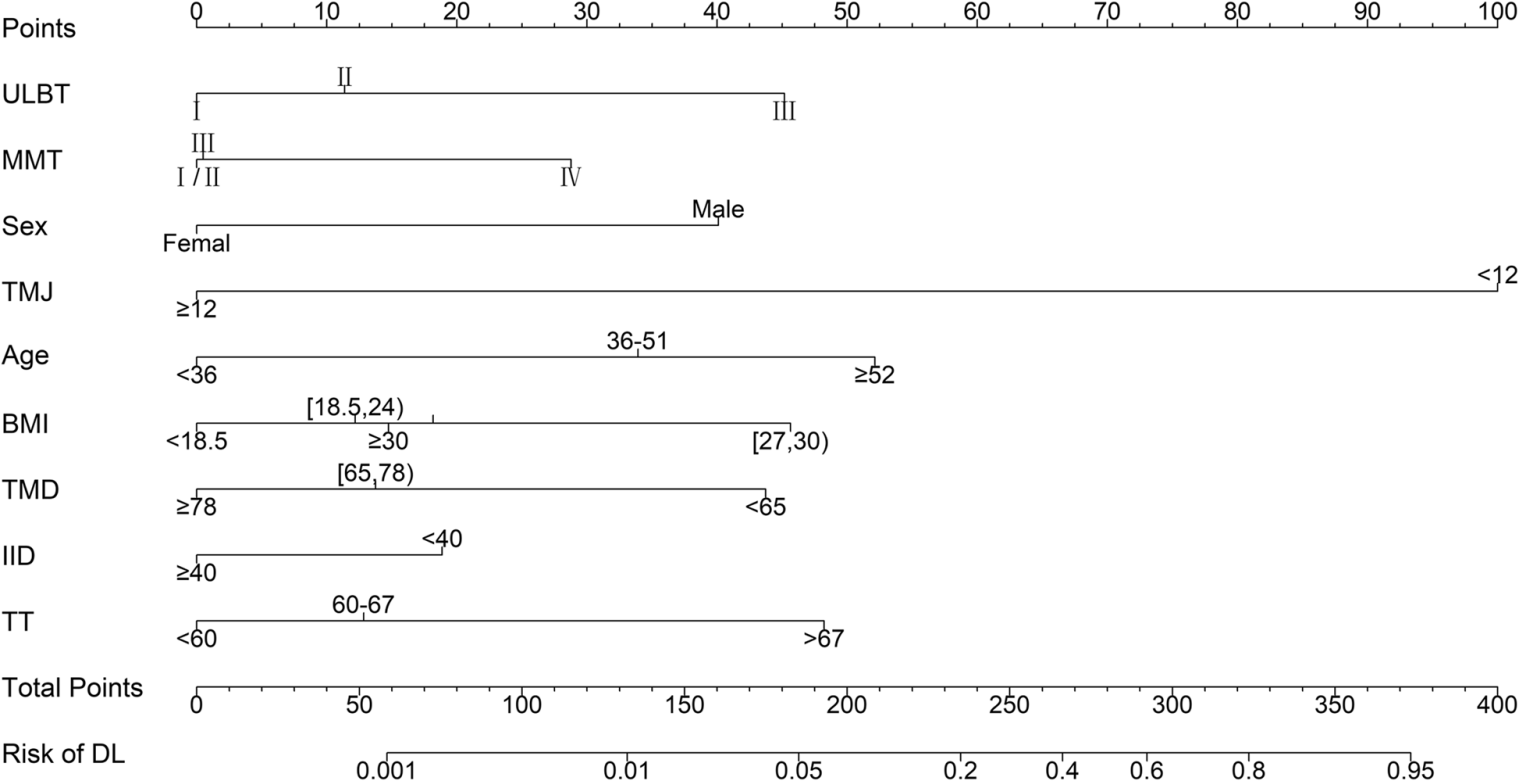
Nomogram predicting difficult laryngoscopy in patients underwent general anesthesia tracheal intubation. Abbreviations: ULBT, upper lip bite test; MMT, modified Mallampati test; TMJ, temporomandibular joint mobility by mandibular condylar mobility measured by ultrasonography; BMI, body mass index; TMD, thyromental distance; IID, interincisor distance; TT, tongue thickness measured by ultrasonography; DL, difficult laryngoscopy

**Fig. 2:**
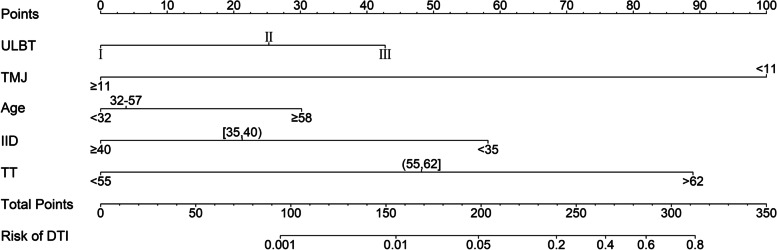
Nomogram predicting difficult tracheal intubation in patients underwent general anesthesia tracheal intubation. Abbreviations: ULBT, upper lip bite test; MMT, modified Mallampati test; TMJ, temporomandibular joint mobility by mandibular condylar mobility measured by ultrasonography; IID, interincisor distance; TT, tongue thickness measured by ultrasonography; DTI, difficult tracheal intubation

**Fig. 3:**
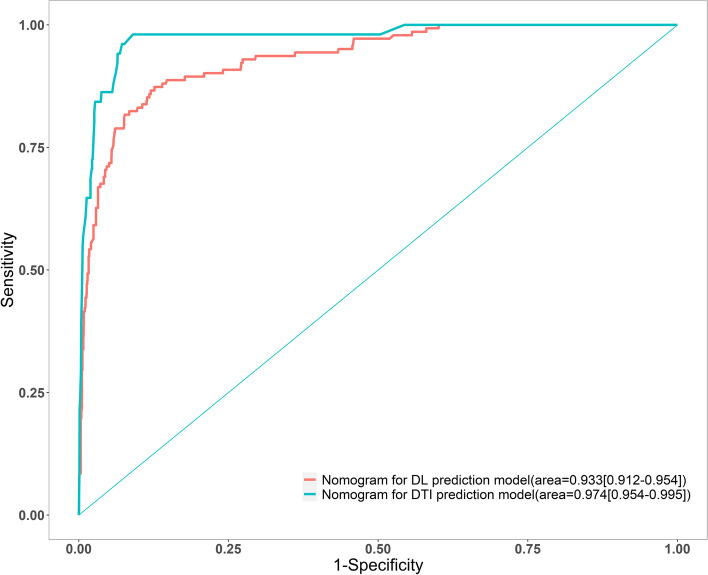
The areas under the receiver operating characteristic curves (AUCs) of nomograms for difficult laryngoscopy and difficult tracheal intubation

The nomogram to predict DL was created based on the following 9 independent factors (Fig. 1): ULBT grade (I, II or III), MMT grade (I/II, III or IV), sex, TMJ (< 12 or ≥ 12 mm), age (< 36, 36–51, ≥ 52 y), BMI (< 18.5, 18.5–24, 24–27, 27–30, ≥ 30), TMD (< 65, 65–78, ≥ 78 mm), IID (< 40, ≥ 40 mm), and TT (< 60, 60–67, ≥ 67 mm). Nomogram for DTI prediction was shown in Fig. 3, including the 5 independent factors: ULBT grade (I, II or III), TMJ (< 11, ≥ 11 mm), age (< 32, 32–57, ≥ 58 y), IID (< 36, 35–40, ≥ 40 mm), and TT (< 55, 55–62, > 62 mm). Score cards of the DL and DTI was shown in Table S6. The higher total points obtained from the sum of the points for each factor in the nomogram means the greater possibility of DL or DTI. For example, a 60 years old (≥ 58 y) patient with ULBT grade II, TMJ 10 mm (< 11 mm), IID 42 mm (≥ 40 mm), TT 65 mm (> 62 mm) was given a total of 244 points for DTI (30 points for age, 25 points for ULBT grade II, 100 points for TMJ, 0 points for IID, and 89 points for TT), thus the predicted DTI risk was about 20% (Fig. 2, Table S6).

To verify the performance of the model, the ROC curve was constructed. The areas under the ROC curves were 0.933 (95% CI: 0.912–0.954) and 0.974 (95% CI: 0.954–0.995) for DL and DTI, respectively (Fig. 3, Table S7).

## Discussion

In this study, nomograms were developed to predict the risk of DL and DTI. These nomograms were constructed by combination of traditional DA assessment methods (such as ULBT, MMT, IID) and ultrasound airway assessment methods (such as TMJ, TT), which have achieved good performance with high AUC values. To our knowledge, this study is the first report that combines traditional DA assessment and ultrasonography-guided airway assessment by nomograms to predict DA.

In our nomogram models, the DL prediction nomogram incorporated 9 variables (ULBT, MMT, sex, TMJ, age, BMI, TMD, IID and TT), and the DTI prediction nomogram included 5 variables (ULBT, TMJ, age, IID and TT). Among these variables, ULBT, MMT, sex, age, BMI, TMD, IID were traditional DA assessment methods while TMJ and TT were ultrasonography-assisted airway assessment methods. In both DL and DTI nomograms, the performance of ultrasonography-assisted airway assessments was good. For example, TT has high nomogram scores in both DL (> 67 mm, 48 points) and DTI (> 62 mm, 89 points) prediction. In particular, TMJ has the highest scores in both DL and DTI nomograms. Our previous study [19, 20] found that TMJ and TT measured by ultrasound can be used to predict DA. TMJ measured by ultrasound performs better predication for DL, compared to traditional DA assessment methods such as IID, ULBT, TMD and MMT. However, due to small sample size (484 patients with 41 DL cases), the performance of TMJ to predict DTI was not assessed [19]. In current study, we showed that TMJ has the highest nomogram scores in both DL (< 12 mm, 100 points) and DTI (< 11 mm, 100 points) prediction, confirming the predictive performance for DA. In a previous study, we have shown that TT is another useful DA predictor [20]. Taken together, our findings indicated that the ultrasonography-assisted DA prediction methods are effective. Hence, we incorporated the TMJ and TT indicators measurement by ultrasound into the nomograms for DL and DTI prediction.

Many studies have attempted to combine indicators to improve predictive capability of DA. Most of these models were created by simple addition of indicators, such as the “3–3-2” rule [21], LEMON criteria [22], and “Wilson” scores [23]. In consistence with these studies, previously we found that the 3–3 rule (IID less than three fingers, a hyoidmental distance less than three fingers) is useful for DA prediction, with AUC 0.709 for DL and 0.822 for DTI [31]. In addition, by using the ratio of TT to TMD, the AUCs for DL and DTI could be significantly improved to 0.75 (95%CI, 0.73–0.76) and 0.86 (95%CI, 0.84–0.87), which are better than that of TT, TMD and MMT alone [20]. In current study, when nomograms were used, the AUCs were increased to 0.933 [95% CI, 0.912–0.954] and 0.974 (95% CI, 0.954–0.995) for DL and DTI, respectively. These data indicated that the nomograms are more powerful tools to predict DA.

The nomograms in this study are easy to implement in routinely clinical practice. As long as nine predictors (ULBT, MMT, Sex, TMJ, Age, BMI, TMD, IID, and TT) were collected, the incidence of DTI and DL in patients could be assessed by the nomograms. Among the nine predictive indicators, ULBT, MMT, Sex, Age, BMI, TMD and IID are all classic indicators commonly used in clinical practice, and the data are very easy to obtain. Furthermore, TT and TMJ can be well measured by conventional clinical ultrasound equipment, and previous studies have shown that the reliability of measurements between different sonographers is comparable [19]. In the actual use of the nomogram, we only need to convert the corresponding predictor value into the corresponding nomogram score value, and then add the score values to obtain the total score. Then the risk incidence corresponding to the total score is obtained, as described in the results section. The operation of nomogram is simple and intuitive, does not require complicated calculation, is less time-consuming, is very convenient to use, and can be quickly popularized.

This study has some limitations. Firstly, data were from a single center and the sample size of DTI and DL patients was relatively small. Secondly, as the patients in this work were all Asian, potential bias and influencing factors must be considered when the models are used for patients in Western countries. Therefore, multicenter, multiracial studies, especially international multicenter research are needed. Thirdly, the ultrasound measurement method adopted in this study may have certain obstacles in clinical application, and not every hospital has the conditions for ultrasound assessment of DA. Finally, more streamlined and efficient methods, and more advanced algorithms are needed to improve the models further.

## Conclusions

Nomograms based on ultrasound airway assessment effectively predicted DL and DTI in patients. The nomogram is an ideal model for DA prediction which may be helpful for patients’ safety who underwent general anesthesia and tracheal intubation surgery.

## Supplementary Information

**Additional file 1: Table S1**. Patients characteristics of difficult laryngoscopy and difficult tracheal intubation.

**Additional file 2: Table S2**. Distribution of continuous variables in difficult laryngoscopy (DL).

**Additional file 3: Table S3**. Distribution of continuous variables in difficult tracheal intubation (DTI).

**Additional file 4: Table S4**. Univariate logistic regression of difficult laryngoscopy (DL) according to the restricted cubic spline (RCS).

**Additional file 5: Table S5**. Univariate logistic regression of difficult tracheal intubation (DTI) according to the restricted cubic spline (RCS).

**Additional file 6: Table S6**. Score cards of the difficult laryngoscopy and difficult tracheal intubation.

**Additional file 7: Table S7**. Performance of the difficult laryngoscopy and difficult tracheal intubation nomogram for prediction models.

**Additional file 8: Figure S1**. The restricted cubic spline (RCS) of TMJ in DL.

**Additional file 9: Figure S2**. The restricted cubic spline (RCS) of age in DL.

**Additional file 10: Figure S3**. The restricted cubic spline (RCS) of TMD in DL.

**Additional file 11: Figure S4**. The restricted cubic spline (RCS) of IID in DL.

**Additional file 12: Figure S5**. The restricted cubic spline (RCS) of TT in DL.

**Additional file 13: Figure S6**. The restricted cubic spline (RCS) of age in DTI.

**Additional file 14: Figure S7**. The restricted cubic spline (RCS) of TMD in DTI.

**Additional file 15: Figure S8**. The restricted cubic spline (RCS) of IID in DTI.

**Additional file 16: Figure S9**. The restricted cubic spline (RCS) of TT in DL.

## Data Availability

The datasets used and analyzed during this current study are available from the corresponding author on reasonable request.

## Abbreviations

AUC: Area under the receiver operating characteristic curve
CI: Confdence interval
DA: Difficult airway
DTI: Difficult tracheal intubation
DL: Difficult laryngoscopy
ULBT: Upper lip bite test
MMT: Modified Mallampati test
TMD: Thyromental distance
IID: Interincisor distance
TMJ: Temporomandibular joint
TT: Tongue thickness
ASA: American Society of Anesthesiologists
ROC: Receiver operating characteristic
AIC: Akaike information criterion
RCS: Restricted cubic spline
OR: Odds ratio

## References

[1] Domino KB (2021). Death and brain damage from difficult airway management: a "never event". Can J Anaesth.

[2] Evans A, Morton B, Groom P (2020). Difficult airway society guidelines for awake tracheal intubation in adults - is lidocaine topicalisation safe?. Anaesthesia..

[3] Goyal T, Bhoi D, Bulbul J (2021). Airway management in a child with an aggressive nasopharyngeal tumor: a challenge for the anesthesiologist. J Clin Anesth.

[4] Heidegger T (2021). Management of the difficult airway. N Engl J Med.

[5] Kornas RL, Owyang CG, Sakles JC, Foley LJ, Mosier JM (2021). Society for Airway Management's special projects C: evaluation and management of the physiologically difficult airway: consensus recommendations from society for airway management. Anesth Analg.

[6] McNarry AF, Asai T (2021). New evidence to inform decisions and guidelines in difficult airway management. Br J Anaesth.

[7] Apfelbaum JL, Hagberg CA, Caplan RA, Blitt CD, Connis RT, Nickinovich DG (2013). Practice guidelines for management of the difficult airway: an updated report by the American society of anesthesiologists task force on management of the difficult airway. Anesthesiology..

[8] Cook F, Lobo D, Martin M, Imbert N, Grati H, Daami N (2019). Prospective validation of a new airway management algorithm and predictive features of intubation difficulty. Br J Anaesth.

[9] Fan S, Chan A, Au S, Leong MC, Chow M, Fan YT (2018). Personalised anaesthesia: three-dimensional printing of facial prosthetic for facial deformity with difficult airway. Br J Anaesth.

[10] Frerk C, Mitchell VS, McNarry AF, Mendonca C, Bhagrath R, Patel A (2015). Difficult airway society 2015 guidelines for management of unanticipated difficult intubation in adults. Br J Anaesth.

[11] Stratton J, Broadhurst P, Jackson M (2018). A team approach to the difficult airway. Br J Anaesth.

[12] Detsky ME, Jivraj N, Adhikari NK, Friedrich JO, Pinto R, Simel DL (2019). Will this patient be difficult to intubate?: the rational clinical examination systematic review. JAMA..

[13] Lundstrom LH, Vester-Andersen M, Moller AM, Charuluxananan S, L'Hermite J, Wetterslev J (2011). Poor prognostic value of the modified Mallampati score: a meta-analysis involving 177 088 patients. Br J Anaesth.

[14] Yildiz TS, Korkmaz F, Solak M, Toker K, Erciyes N, Bayrak F (2007). Prediction of difficult tracheal intubation in Turkish patients: a multi-center methodological study. Eur J Anaesthesiol.

[15] Adi O, Fong CP, Sum KM, Ahmad AH (2021). Usage of airway ultrasound as an assessment and prediction tool of a difficult airway management. Am J Emerg Med.

[16] Austin DR, Chang MG, Bittner EA (2021). Use of handheld point-of-care ultrasound in emergency airway management. Chest..

[17] Bianchini A, Nardozi L, Nardi E, Scuppa MF (2021). Airways ultrasound in predicting difficult face mask ventilation. Minerva Anestesiol.

[18] Ni H, Guan C, He G, Bao Y, Shi D, Zhu Y (2020). Ultrasound measurement of laryngeal structures in the parasagittal plane for the prediction of difficult laryngoscopies in Chinese adults. BMC Anesthesiol.

[19] Yao W, Zhou Y, Wang B, Yu T, Shen Z, Wu H (2017). Can mandibular condylar mobility sonography measurements predict difficult laryngoscopy?. Anesth Analg.

[20] Yao W, Wang B (2017). Can tongue thickness measured by ultrasonography predict difficult tracheal intubation?. Br J Anaesth.

[21] Mahmoodpoor A, Soleimanpour H, Nia KS, Panahi JR, Afhami M, Golzari SE (2013). Sensitivity of palm print, modified mallampati score and 3-3-2 rule in prediction of difficult intubation. Int J Prev Med.

[22] Hagiwara Y, Watase H, Okamoto H, Goto T, Hasegawa K (2015). Japanese emergency medicine network I: prospective validation of the modified LEMON criteria to predict difficult intubation in the ED. Am J Emerg Med.

[23] Wilson ME, Spiegelhalter D, Robertson JA, Lesser P (1988). Predicting difficult intubation. Br J Anaesth.

[24] Chen D, Chen G, Jiang W, Fu M, Liu W, Sui J (2019). Association of the collagen signature in the tumor microenvironment with lymph node metastasis in early gastric cancer. JAMA Surg.

[25] Chen D, Liu Z, Liu W, Fu M, Jiang W, Xu S (2021). Predicting postoperative peritoneal metastasis in gastric cancer with serosal invasion using a collagen nomogram. Nat Commun.

[26] Lei Z, Li J, Wu D, Xia Y, Wang Q, Si A (2016). Nomogram for preoperative estimation of microvascular invasion risk in hepatitis B virus-related hepatocellular carcinoma within the Milan criteria. JAMA Surg..

[27] Odonkor CA, Christiansen S, Chen Y, Sathiyakumar A, Chaudhry H, Cinquegrana D (2017). Factors associated with missed appointments at an academic pain treatment center: a prospective year-long longitudinal study. Anesth Analg.

[28] Zhang LL, Xu F, Song D, Huang MY, Huang YS, Deng QL (2020). Development of a nomogram model for treatment of nonmetastatic nasopharyngeal carcinoma. JAMA Netw Open.

[29] Wang B, Zheng C, Yao W, Guo L, Peng H, Yang F (2019). Predictors of difficult airway in a Chinese surgical population: the gender effect. Minerva Anestesiol.

[30] Cormack RS, Lehane J (1984). Difficult tracheal intubation in obstetrics. Anaesthesia..

[31] Yu T, Wang B, Jin XJ, Wu RR, Wu H, He JJ (2015). Predicting difficult airways: 3-3-2 rule or 3-3 rule?. Ir J Med Sci.

