# Supplementary material for: Nomograms for predicting difficult airway based on ultrasound assessment
Source: BMC Anesthesiol. 2022 Jan 13;22:23. doi: 10.1186/s12871-022-01567-y (PMC8756724; doi:10.1186/s12871-022-01567-y)
Supplement: Supplementary file 1 — Additional file 1: Table S1. Patients characteristics of difficult laryngoscopy and difficult tracheal intubation. [file 12871_2022_1567_MOESM1_ESM.docx]

| Variable | Total (n=2254) | Difficult laryngoscopy (DL) | | |  | Difficult tracheal intubation (DTI) | | |
| --- | --- | --- | --- | --- | --- | --- | --- | --- |
|  |  | Non-DL (n=2112) | DL (n=142) | *P* |  | Non-DTI (n=2203) | DTI (n=51) | *P* |
| ULBT |  |  |  | <0.001 |  |  |  | <0.001 |
| Ⅰ | 876 (38.86) | 853 (97.37) | 23 (2.63) |  |  | 872 (99.54) | 4 (0.46) |  |
| Ⅱ | 1210 (53.68) | 1138 (94.05) | 72 (5.95) |  |  | 1184 (97.85) | 26 (2.15) |  |
| Ⅲ | 168 (7.45) | 121 (72.02) | 47 (27.98) |  |  | 147 (87.50) | 21 (12.50) |  |
| MMT |  |  |  | <0.001 |  |  |  | <0.001 |
| Ⅰ/Ⅱ | 1245 (55.24) | 1201 (96.47) | 44 (3.53) |  |  | 1231 (98.88) | 14 (1.12) |  |
| Ⅲ | 617 (27.37) | 576 (93.35) | 41 (6.65) |  |  | 605 (98.06) | 12 (1.94) |  |
| Ⅳ | 392 (17.39) | 335 (85.46) | 57 (14.54) |  |  | 367 (93.62) | 25 (6.38) |  |
| Sex |  |  |  | <0.001 |  |  |  | 0.004 |
| Male | 1059 (46.98) | 957 (90.37) | 102 (9.63) |  |  | 1025 (96.79) | 34 (3.21) |  |
| Female | 1195 (53.02) | 1155 (96.65) | 40 (3.35) |  |  | 1178 (98.58) | 17 (1.42) |  |
| TMJ | 13.00 (12.00, 15.00) | 13.00 (12.00, 15.00) | 10.00 (8.00, 11.00) | <0.001 |  | 13.00 (12.00, 15.00) | 8.00 (7.00, 9.00) | <0.001 |
| Age | 50.00 (42.00, 62.00) | 49.00 (41.00, 61.00) | 61.00 (50.00, 67.75) | <0.001 |  | 50.00 (42.00, 62.00) | 61.00 (52.00, 69.50) | <0.001 |
| BMI | 22.50 (20.45, 24.84) | 22.49 (20.45, 24.78) | 23.12 (20.86, 25.37) | 0.0604 |  | 22.50 (20.45, 24.84) | 23.18 (21.75, 26.70) | 0.084 |
| TMD | 73.00 (69.00, 80.00) | 73.00 (70.00, 80.00) | 69.00 (64.00, 74.00) | <0.001 |  | 73.00 (69.00, 80.00) | 68.00 (64.50, 70.00) | <0.001 |
| IID | 41.00 (37.00, 45.00) | 41.00 (38.00, 45.00) | 36.00 (31.25, 40.00) | <0.001 |  | 41.00 (37.00, 45.00) | 32.00 (29.50, 35.50) | <0.001 |
| TT | 59.00 (55.00, 62.00) | 58.00 (55.00, 62.00) | 62.00 (58.25, 66.00) | <0.001 |  | 59.00 (55.00, 62.00) | 64.00 (61.50, 66.50) | <0.001 |
