# Supplementary material for: Nomograms for predicting difficult airway based on ultrasound assessment
Source: BMC Anesthesiol. 2022 Jan 13;22:23. doi: 10.1186/s12871-022-01567-y (PMC8756724; doi:10.1186/s12871-022-01567-y)
Supplement: Supplementary file 2 — Additional file 2: Table S2. Distribution of continuous variables in difficult laryngoscopy (DL). [file 12871_2022_1567_MOESM2_ESM.docx]

|  | | P5 | P25 | P75 | P95 |
| --- | --- | --- | --- | --- | --- |
| Non-DL | TMJ | 10 | 12 | 15 | 17 |
|  | Age | 23 | 41 | 61 | 73 |
|  | BMI | 17.8 | 20.4 | 24.8 | 29.1 |
|  | TMD | 62 | 70 | 80 | 90 |
|  | IID | 32 | 38 | 45 | 50 |
|  | TT | 50 | 55 | 62 | 67 |
| DL | TMJ | 6 | 8 | 11 | 13 |
|  | Age | 43 | 50 | 68 | 76 |
|  | BMI | 18.1 | 20.8 | 25.4 | 28.1 |
|  | TMD | 59 | 64 | 74 | 88 |
|  | IID | 25 | 31 | 40 | 44 |
|  | TT | 54 | 58 | 66 | 70 |
