# Supplementary material for: Nomograms for predicting difficult airway based on ultrasound assessment
Source: BMC Anesthesiol. 2022 Jan 13;22:23. doi: 10.1186/s12871-022-01567-y (PMC8756724; doi:10.1186/s12871-022-01567-y)
Supplement: Supplementary file 3 — Additional file 3: Table S3. Distribution of continuous variables in difficult tracheal intubation (DTI). [file 12871_2022_1567_MOESM3_ESM.docx]

|  | | P5 | P25 | P75 | P95 |
| --- | --- | --- | --- | --- | --- |
| Non-DTI | TJM | 10 | 12 | 15 | 17 |
|  | Age | 23 | 42 | 62 | 73 |
|  | BMI | 17.8 | 20.4 | 24.8 | 29.0 |
|  | TMD | 61 | 69 | 80 | 90 |
|  | IID | 31 | 37 | 45 | 50 |
|  | TT | 50 | 55 | 62 | 67 |
| DTI | TJM | 5 | 7 | 9 | 11 |
|  | Age | 44 | 51 | 70 | 77 |
|  | BMI | 17.6 | 21.7 | 27.0 | 31.2 |
|  | TMD | 59 | 64 | 70 | 79 |
|  | IID | 22 | 29 | 36 | 40 |
|  | TT | 56 | 61 | 67 | 69 |
