# Supplementary material for: Nomograms for predicting difficult airway based on ultrasound assessment
Source: BMC Anesthesiol. 2022 Jan 13;22:23. doi: 10.1186/s12871-022-01567-y (PMC8756724; doi:10.1186/s12871-022-01567-y)
Supplement: Supplementary file 4 — Additional file 4: Table S4. Univariate logistic regression of difficult laryngoscopy (DL) according to the restricted cubic spline (RCS). [file 12871_2022_1567_MOESM4_ESM.docx]

|  | Total (n=2254) | No-DL (n=2112) | DL (n=142) | Statistic value | *P* | Test method |
| --- | --- | --- | --- | --- | --- | --- |
| ULBT |  |  |  | 154.008 | <0.001 | Pearson χ^2^ |
| Ⅰ | 876 (38.86) | 853 (97.37) | 23 (2.63) |  | a |  |
| Ⅱ | 1210 (53.68) | 1138 (94.05) | 72 (5.95) |  | b |  |
| Ⅲ | 168 (7.45) | 121 (72.02) | 47 (27.98) |  | c |  |
| MMT |  |  |  | 61.356 | <0.001 | Pearson χ^2^ |
| Ⅰ/Ⅱ | 1245 (55.24) | 1201 (96.47) | 44 (3.53) |  | a |  |
| Ⅲ | 617 (27.37) | 576 (93.35) | 41 (6.65) |  | b |  |
| Ⅳ | 392 (17.39) | 335 (85.46) | 57 (14.54) |  | c |  |
| Sex |  |  |  | 37.564 | <0.001 | Pearson χ^2^ |
| Male | 1059 (46.98) | 957 (90.37) | 102 (9.63) |  |  |  |
| Female | 1195 (53.02) | 1155 (96.65) | 40 (3.35) |  |  |  |
| TMJ |  |  |  | 383.149 | <0.001 | Pearson χ^2^ |
| <12 | 490 (21.74) | 366 (74.69) | 124 (25.31) |  |  |  |
| ≥12 | 1764 (78.26) | 1746 (98.98) | 18 (1.02) |  |  |  |
| Age |  |  |  | 46.379 | <0.001 | Pearson χ^2^ |
| <36 | 336 (14.91) | 333 (99.11) | 3 (0.89) |  | a |  |
| 36-51 | 918 (40.73) | 879 (95.75) | 39 (4.25) |  | b |  |
| ≥52 | 1000 (44.37) | 900 (90.00) | 100 (10.00) |  | c |  |
| BMI |  |  |  | 9.688 | 0.046 | Pearson χ^2^ |
| <18.5 | 211 (9.36) | 199 (94.31) | 12 (5.69) |  | ab |  |
| [18.5,24) | 1277 (56.65) | 1208 (94.60) | 69 (5.40) |  | a |  |
| [24,27) | 500 (22.18) | 463 (92.60) | 37 (7.40) |  | ab |  |
| [27-30) | 197 (8.74) | 176 (89.34) | 21 (10.66) |  | b |  |
| ≥30 | 69 (3.06) | 66 (95.65) | 3 (4.35) |  | ab |  |
| TMD |  |  |  | 68.806 | <0.001 | Pearson χ^2^ |
| <65 | 219 (9.72) | 178 (81.28) | 41 (18.72) |  | a |  |
| [65,78) | 1262 (55.99) | 1187 (94.06) | 75 (5.94) |  | b |  |
| ≥78 | 773 (34.29) | 747 (96.64) | 26 (3.36) |  | c |  |
| IID |  |  |  | 92.126 | <0.001 | Pearson χ^2^ |
| <40 | 821 (36.42) | 716 (87.21) | 105 (12.79) |  |  |  |
| ≥40 | 1433 (63.58) | 1396 (97.42) | 37 (2.58) |  |  |  |
| TT |  |  |  | 58.204 | <0.001 | Pearson χ^2^ |
| <60 | 1283 (56.92) | 1237 (96.41) | 46 (3.59) |  | a |  |
| 60-67 | 874 (38.78) | 798 (91.30) | 76 (8.70) |  | b |  |
| >67 | 97 (4.30) | 77 (79.38) | 20 (20.62) |  | c |  |
