# Supplementary material for: Nomograms for predicting difficult airway based on ultrasound assessment
Source: BMC Anesthesiol. 2022 Jan 13;22:23. doi: 10.1186/s12871-022-01567-y (PMC8756724; doi:10.1186/s12871-022-01567-y)
Supplement: Supplementary file 5 — Additional file 5: Table S5. Univariate logistic regression of difficult tracheal intubation (DTI) according to the restricted cubic spline (RCS). [file 12871_2022_1567_MOESM5_ESM.docx]

|  | Total (n=2254) | No-DTI (n=2203) | DTI (n=51) | Statistic value | *P* | Test method |
| --- | --- | --- | --- | --- | --- | --- |
| ULBT |  |  |  | 92.609 | <0.001 | Pearson χ^2^ |
| Ⅰ | 876 (38.86) | 872 (99.54) | 4 (0.46) |  | a |  |
| Ⅱ | 1210 (53.68) | 1184 (97.85) | 26 (2.15) |  | b |  |
| Ⅲ | 168 (7.45) | 147 (87.50) | 21 (12.50) |  | c |  |
| MMT |  |  |  | 37.589 | <0.001 | Pearson χ^2^ |
| Ⅰ/Ⅱ | 1245 (55.24) | 1231 (98.88) | 14 (1.12) |  | a |  |
| Ⅲ | 617 (27.37) | 605 (98.06) | 12 (1.94) |  | a |  |
| Ⅳ | 392 (17.39) | 367 (93.62) | 25 (6.38) |  | b |  |
| Sex |  |  |  | 8.116 | 0.00439 | Pearson χ^2^ |
| Male | 1059 (46.98) | 1025 (96.79) | 34 (3.21) |  |  |  |
| Female | 1195 (53.02) | 1178 (98.58) | 17 (1.42) |  |  |  |
| TMJ |  |  |  | 390.070 | <0.001 | Continuous χ^2^ correction |
| <11 | 212 (9.41) | 166 (78.30) | 46 (21.70) |  |  |  |
| ≥11 | 2042 (90.59) | 2037 (99.76) | 5 (0.24) |  |  |  |
| Age |  |  |  | 24.876 | <0.001 | Pearson χ^2^ |
| <32 | 251 (11.14) | 250 (99.60) | 1 (0.40) |  | a |  |
| 32-58 | 1228 (54.48) | 1212 (98.70) | 16 (1.30) |  | a |  |
| ≥58 | 775 (34.38) | 741 (95.61) | 34 (4.39) |  | b |  |
| BMI |  |  |  |  | 0.116 | Fisher |
| <18.5 | 211 (9.36) | 206 (97.63) | 5 (2.37) |  |  |  |
| [18.5,24) | 1277 (56.65) | 1253 (98.12) | 24 (1.88) |  |  |  |
| [24,27) | 500 (22.18) | 490 (98.00) | 10 (2.00) |  |  |  |
| [27-30) | 197 (8.74) | 188 (95.43) | 9 (4.57) |  |  |  |
| ≥30 | 69 (3.06) | 66 (95.65) | 3 (4.35) |  |  |  |
| TMD |  |  |  | 13.019 | <0.001 | Continuous χ^2^ correction |
| <65 | 219 (9.72) | 206 (94.06) | 13 (5.94) |  |  |  |
| ≥65 | 2035 (90.28) | 1997 (98.13) | 38 (1.87) |  |  |  |
| IID |  |  |  | 132.290 | <0.001 | Pearson χ^2^ |
| <35 | 274 (12.16) | 242 (10.99) | 32 (62.75) |  | a |  |
| [35,40) | 682 (30.26) | 667 (30.28) | 15 (29.41) |  | b |  |
| ≥40 | 1298 (57.59) | 1294 (58.74) | 4 (7.84) |  | c |  |
| TT |  |  |  | 55.104 | <0.001 | Pearson χ^2^ |
| ≤55 | 615 (27.28) | 613 (27.83) | 2 (3.92) |  | a |  |
| (55,62] | 1130 (50.13) | 1114 (50.57) | 16 (31.37) |  | b |  |
| >62 | 509 (22.58) | 476 (21.61) | 33 (64.71) |  | c |  |
