# Supplementary material for: Nomograms for predicting difficult airway based on ultrasound assessment
Source: BMC Anesthesiol. 2022 Jan 13;22:23. doi: 10.1186/s12871-022-01567-y (PMC8756724; doi:10.1186/s12871-022-01567-y)
Supplement: Supplementary file 6 — Additional file 6: Table S6. Score cards of the difficult laryngoscopy and difficult tracheal intubation. [file 12871_2022_1567_MOESM6_ESM.docx]

| Nomogram for DL prediction model | |  | Nomogram for DTI prediction model | |
| --- | --- | --- | --- | --- |
| Variable | Point |  | Variable | Point |
| ULBT |  |  | ULBT |  |
| Ⅰ | 0 |  | Ⅰ | 0 |
| Ⅱ | 11 |  | Ⅱ | 25 |
| Ⅲ | 45 |  | Ⅲ | 43 |
| MMT |  |  |  |  |
| Ⅰ/Ⅱ | 0 |  |  |  |
| Ⅲ | 0 |  |  |  |
| Ⅳ | 29 |  |  |  |
| Sex |  |  |  |  |
| Male | 40 |  |  |  |
| Female | 0 |  |  |  |
| TMJ |  |  | TMJ |  |
| <12 | 100 |  | <11 | 100 |
| ≥12 | 0 |  | ≥11 | 0 |
| Age |  |  | Age |  |
| <36 | 0 |  | <32 | 0 |
| 36-51 | 34 |  | 32-58 | 4 |
| ≥52 | 52 |  | ≥58 | 30 |
| BMI |  |  |  |  |
| <18.5 | 0 |  |  |  |
| [18.5,24) | 12 |  |  |  |
| [24,27) | 18 |  |  |  |
| [27-30) | 46 |  |  |  |
| ≥30 | 15 |  |  |  |
| TMD |  |  |  |  |
| <65 | 44 |  |  |  |
| [65,78) | 14 |  |  |  |
| ≥78 | 0 |  |  |  |
| IID |  |  | IID |  |
| <40 | 19 |  | <35 | 58 |
| ≥40 | 0 |  | [35,40) | 21 |
| TT |  |  | ≥40 | 0 |
| <60 | 0 |  | TT |  |
| 60-67 | 13 |  | ≤55 | 0 |
| >67 | 48 |  | (55,62] | 48 |
|  |  |  | >62 | 89 |
| cutoff=196 | |  | cutoff=164 | |
