# Supplementary material for: Nomograms for predicting difficult airway based on ultrasound assessment
Source: BMC Anesthesiol. 2022 Jan 13;22:23. doi: 10.1186/s12871-022-01567-y (PMC8756724; doi:10.1186/s12871-022-01567-y)
Supplement: Supplementary file 7 — Additional file 7: Table S7. Performance of the difficult laryngoscopy and difficult tracheal intubation nomogram for prediction models. [file 12871_2022_1567_MOESM7_ESM.docx]

|  | cutoff | AUC | sensitivity | Specificity |
| --- | --- | --- | --- | --- |
| Nomogram for DL prediction model | 196 | 0.933(0.912-0.954) | 0.873 | 0.874 |
| Nomogram for DTI prediction model | 164 | 0.974(0.954-0.995) | 0.980 | 0.910 |
