# Supplementary figures and images for: Nomograms for predicting difficult airway based on ultrasound assessment
Source: BMC Anesthesiol. 2022 Jan 13;22:23. doi: 10.1186/s12871-022-01567-y (PMC8756724; doi:10.1186/s12871-022-01567-y)

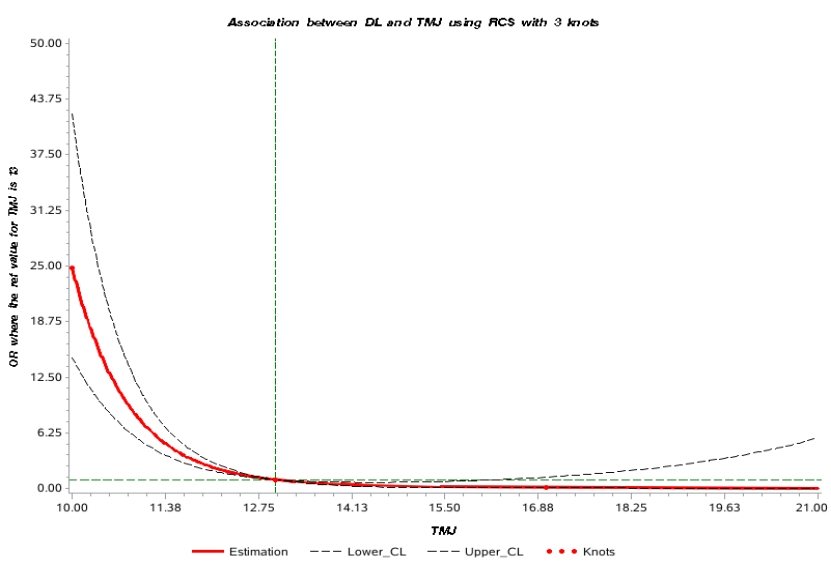

Supplement: Supplementary file 8 — Additional file 8: Figure S1. The restricted cubic spline (RCS) of TMJ in DL. [file 12871_2022_1567_MOESM8_ESM.docx]

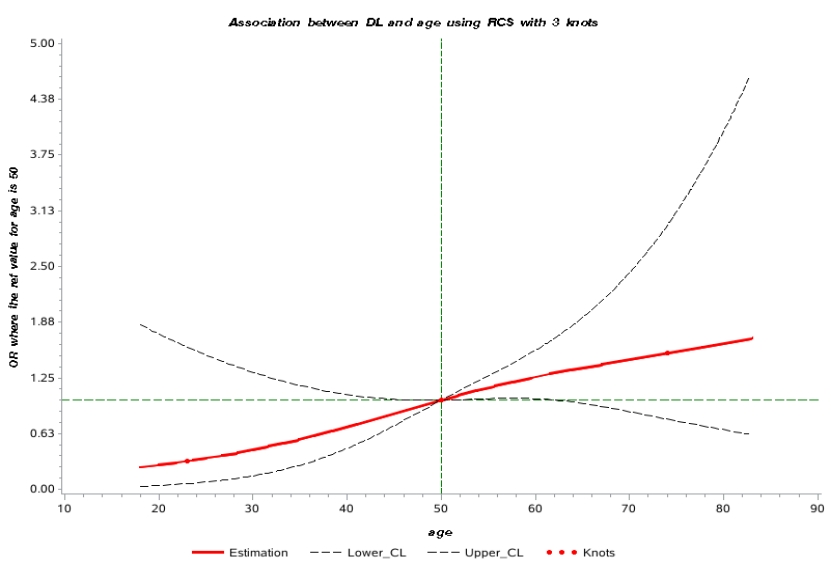

Supplement: Supplementary file 9 — Additional file 9: Figure S2. The restricted cubic spline (RCS) of age in DL. [file 12871_2022_1567_MOESM9_ESM.docx]

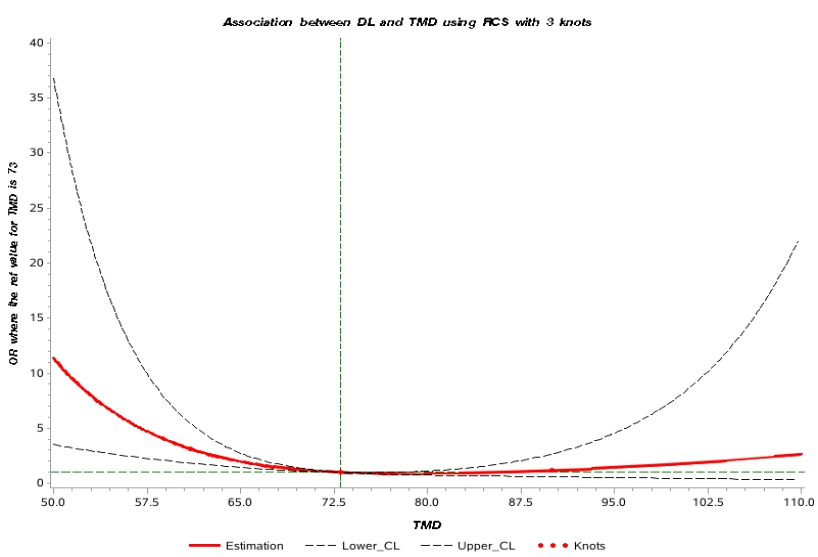

Supplement: Supplementary file 10 — Additional file 10: Figure S3. The restricted cubic spline (RCS) of TMD in DL. [file 12871_2022_1567_MOESM10_ESM.docx]

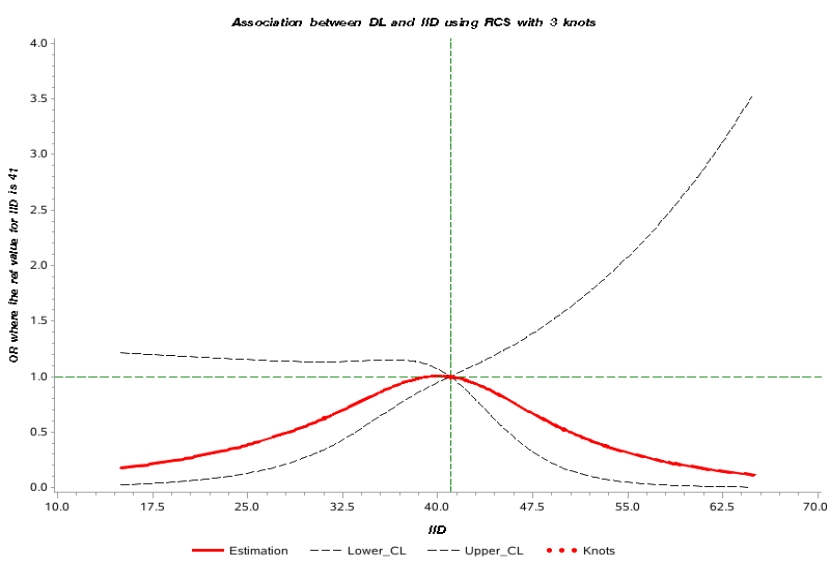

Supplement: Supplementary file 11 — Additional file 11: Figure S4. The restricted cubic spline (RCS) of IID in DL. [file 12871_2022_1567_MOESM11_ESM.docx]

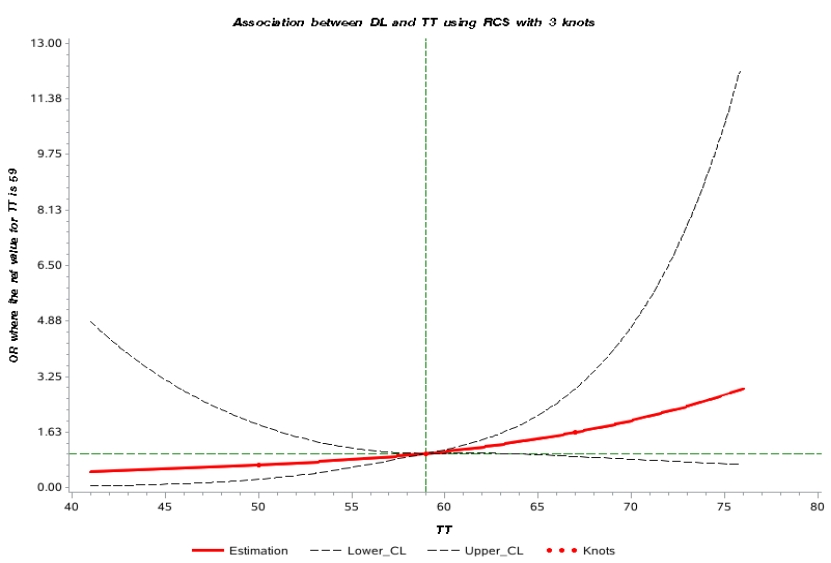

Supplement: Supplementary file 12 — Additional file 12: Figure S5. The restricted cubic spline (RCS) of TT in DL. [file 12871_2022_1567_MOESM12_ESM.docx]

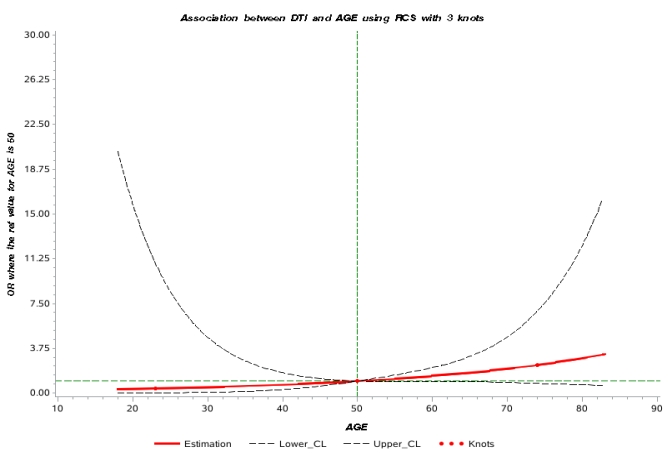

Supplement: Supplementary file 13 — Additional file 13: Figure S6. The restricted cubic spline (RCS) of age in DTI. [file 12871_2022_1567_MOESM13_ESM.docx]

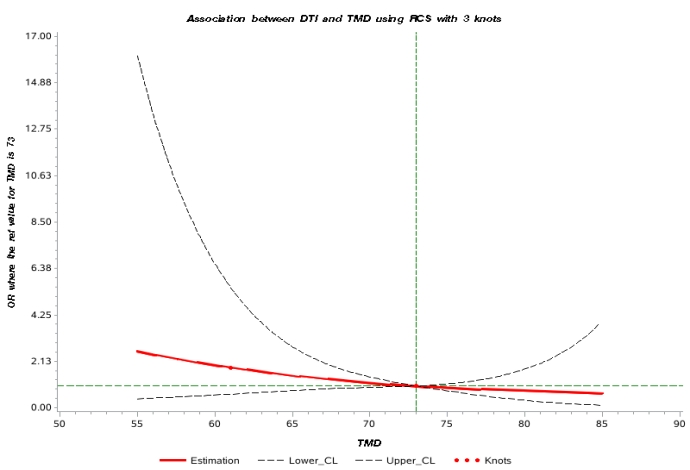

Supplement: Supplementary file 14 — Additional file 14: Figure S7. The restricted cubic spline (RCS) of TMD in DTI. [file 12871_2022_1567_MOESM14_ESM.docx]

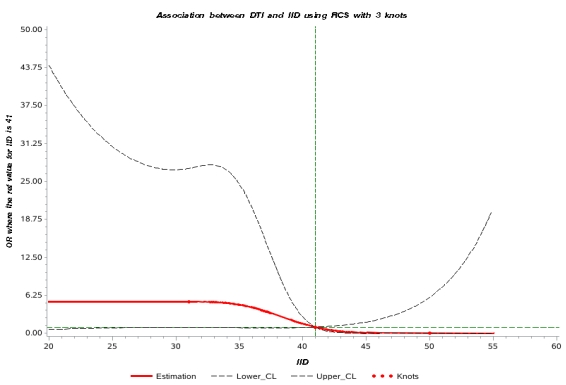

Supplement: Supplementary file 15 — Additional file 15: Figure S8. The restricted cubic spline (RCS) of IID in DTI. [file 12871_2022_1567_MOESM15_ESM.docx]

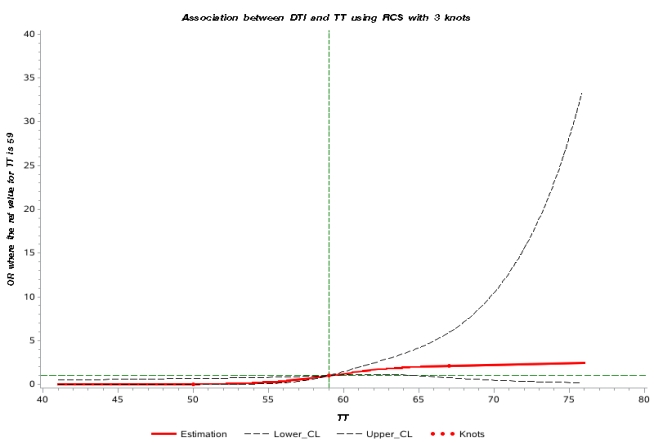

Supplement: Supplementary file 16 — Additional file 16: Figure S9. The restricted cubic spline (RCS) of TT in DL. [file 12871_2022_1567_MOESM16_ESM.docx]
